# Supplementary material for: Chronic ethanol consumption compromises neutrophil function in acute pulmonary Aspergillus fumigatus infection
Source: eLife. 2020 Jul 23;9:e58855. doi: 10.7554/eLife.58855 (PMC7398701; doi:10.7554/eLife.58855)
Supplement: Supplementary file 1. [file elife-58855-supp1.docx]

Supplementary File 1. Percentage of neutrophil precursors in bone marrow.

| Neutrophil  precursor | Groups | | |  |  |
| --- | --- | --- | --- | --- | --- |
|  | Control | Ethanol-treated | | |  |
| Promyelocytes | 0.333± 0.577 | | 0.333± 0.577 | | |
| Myelocytes | 0.000± 0.000 | | 0.000± 0.000 | | |
| Metamyelocytes | 1.167± 0.289 | | 0.833± 1.041 | | |

C57BL/6 mice received ethanol 5% (v/v) in the first week, followed by 10% (v/v) in the second week and treated during 10 weeks with ethanol 20% (v/v) in the drinking water. Control group received water. After ethanol treatment, mice were euthanized and bone marrow (BM) from femurs were collected. Total cell counts were determined by counting in Neubauer chamber and differential neutrophil precursors count was obtained from cytospin preparations. Data are presented as % of cells ± SD (3 mice per group).
